# A novel and compact review on the role of oxidative stress in female reproduction

**DOI:** 10.1186/s12958-018-0391-5

**Published:** 2018-08-20

**Authors:** Jiayin Lu, Zixu Wang, Jing Cao, Yaoxing Chen, Yulan Dong

**Affiliations:** Laboratory of Neurobiology, College of Animal Medicine, China Agricultural University, Haidian, Beijing, 100193 People’s Republic of China

**Keywords:** ROS, Oxidative stress, Reproductive diseases, Antioxidants, Imbalance, Female fertility, Signaling pathways

## Abstract

In recent years, the study of oxidative stress (OS) has become increasingly popular. In particular, the role of OS on female fertility is very important and has been focused on closely. The occurrence of OS is due to the excessive production of reactive oxygen species (ROS). ROS are a double-edged sword; they not only play an important role as secondary messengers in many intracellular signaling cascades, but they also exert indispensable effects on pathological processes involving the female genital tract. ROS and antioxidants join in the regulation of reproductive processes in both animals and humans. Imbalances between pro-oxidants and antioxidants could lead to a number of female reproductive diseases. This review focuses on the mechanism of OS and a series of female reproductive processes, explaining the role of OS in female reproduction and female reproductive diseases caused by OS, including polycystic ovary syndrome (PCOS), endometriosis, preeclampsia and so on. Many signaling pathways involved in female reproduction, including the Keap1-Nrf2, NF-κB, FOXO and MAPK pathways, which are affected by OS, are described, providing new ideas for the mechanism of reproductive diseases.

## Background

Oxygen is a necessary element of aerobic life, and oxidative metabolism represents a principal source of energy. Cells have a defense system against ROS under aerobic conditions, and in healthy biology, there is an appropriate balance between pro-oxidants and antioxidants. OS occurs with the generation of excessive ROS or when the antioxidants’ defense mechanisms are weakened [1–3]. The most important biologically ROS are superoxide anion (O_2_^−^•), hydroxyl radical (•OH), peroxyl (ROO^•^), alkoxyl (RO^•^) and hydroperoxyl (HO_2_^•^). Free radical species are unstable and highly reactive, but they can become stable by acquiring electrons from lipids, nucleic acids, proteins, carbohydrates or nearby molecules, causing a cascade of chain reactions and resulting in cellular damage and disease [4–6]. Therefore, OS can cause DNA damage, lipids peroxidation and protein damage. Under normal circumstances, there are two types of antioxidants in the body: non-enzymatic antioxidants and enzymatic antioxidants. Enzymatic antioxidants include superoxide dismutase (SOD), glutathione peroxidase (GPx), catalase (CAT) and glutathione reductase (GSR), which can cause reduction of H_2_O_2_ to water and alcohol. Non-enzymatic antioxidants are known as synthetic antioxidants or dietary supplements, including vitamin C, vitamin E, β-carotene, selenium, zinc, taurine, glutathione and so on [7].

OS is considered to be responsible for the initiation or development of pathological processes affecting female reproductive processes [8, 9], such as embryonic resorption, recurrent pregnancy loss, preeclampsia, intrauterine growth restriction (IUGR) and fetal death [10]. However, the relationship between ROS-induced OS and diseases is unclear and cannot be adequately investigated in human pregnancies because of self-evident ethical reasons. Therefore, animal models of both normal and disturbed pregnancies are essential for filling these important gaps in our knowledge. The normal level of ROS plays an important regulatory role through various signaling transduction pathways in folliculogenesis, corpus luteum oocyte maturation and feto-placental development [11]. However, ROS can sometimes exert damaging effects when overabundant. They have a close relationship with reproductive events, so tightly controlled ROS generation is an important process. It is one of the central elements of cell signaling, gene expression, maintenance of redox homeostasis and signal transduction pathways involved in cell function, growth, differentiation and death [12]. When keywords were searched in the NCBI and Web of Science databases, there were more than 100,000 articles on reproduction and oxidative stress, but there were only approximately 20,000 articles on the relationship between female reproduction and oxidative stress. There were more than 3000 articles about the mechanism, but there were only approximately 800 articles on uterine and ovarian diseases and oxidative stress. There is very little research on the mechanism of uterine and ovarian diseases and oxidative stress, only 30 articles, and review articles are rare. This review not only sheds light on the mechanism of action of oxidative stress under normal physiological conditions, but it also explores and speculates on the mechanisms of joint reproductive diseases, providing readers with more comprehensive content. The 133 articles selected in this article have a greater impact on the fields of reproduction and stress. By summarizing previous studies, a convincing review is offered.

The previous discussion of reproduction and oxidative stress was limited to individual diseases. This review aims to provide a comprehensive discussion of the role of oxidative stress in female reproduction, and it speculates on new mechanisms of action. This review mainly examines the available evidence for the involvement of cellular ROS-induced OS in pregnancy-related diseases, and it explores the new signaling pathways between OS and female reproduction.

## Reproductive processes

**Fig. 1:**
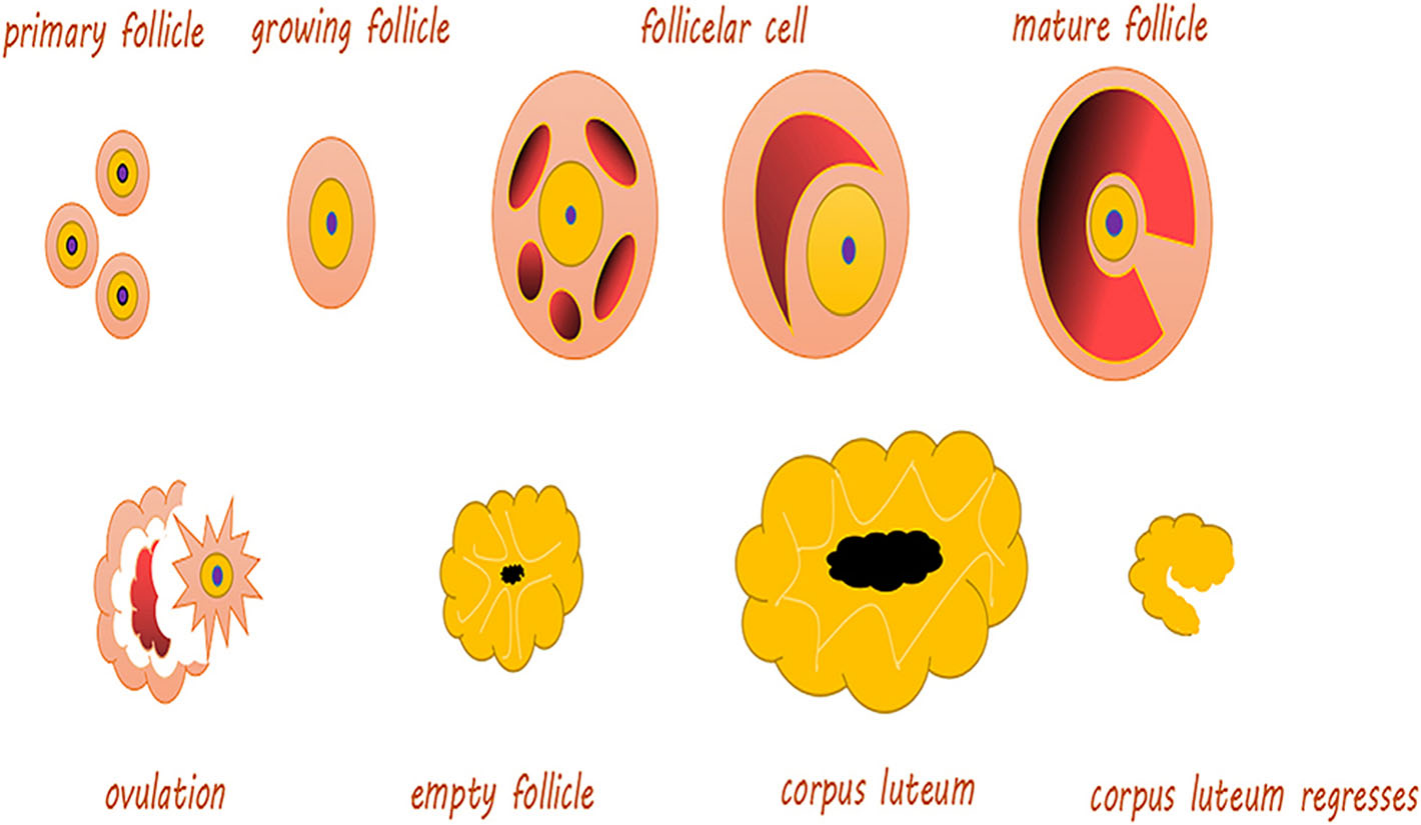
The development of ovarian follicles. Primary follicle: The center has an oocyte, and there is a flat layer of follicular cells on its periphery. Growing follicle: Including the primary growth follicle and secondary growth follicles. Primary growth follicle: One or more layers of cuboidal follicular cells between the egg cells, and follicular cells demonstrate red-stained zona pellucida, while the follicular periphery appears like connective tissue follicular membrane. Secondary growth follicle: Follicular cells appear in the follicular cavity, and some follicular cavities are large, forming a cumulus of oophores. Follicular cells are located on the inner wall of follicles and are arranged in layers, called granular layers. The follicular membrane includes the inner and outer membrane layers. Mature follicle: The follicle cavity is very large, and cumulus oophores are obvious. Follicular endometrial cells appear close to the follicular granule layer. There is a layer of basement membrane between the granule layer cells and follicular endometrial cells; endometrial cells are polygonal, with clear cytoplasm and round nuclei; cells can be seen between many capillaries, and outer membrane cells are located in the outermost layer, mostly spindle shaped with the surrounding connective tissue boundaries not obvious. Ovulation: Mature follicles develop to a certain stage, obviously protruding from the ovarian surface; with the follicular fluid increasing sharply, the pressure increases so that the prominent part of the ovarian tissue becomes thinner and finally ruptures; secondary oocytes and their peripheral zona pellucida and radiation crowns are discharged together with the follicular fluid. Empty follicle: At this time, the follicle is empty, indicating that the corpus luteum starts to form. Corpus luteum: The residual follicle wall collapses after ovulation; the connective tissue of the follicular membrane and capillaries stretches into the particle layer, and as the role of LH evolves, it evolves into a larger volume cell cluster, rich in capillaries and endocrine function and fresh yellow in color

**Fig. 2:**
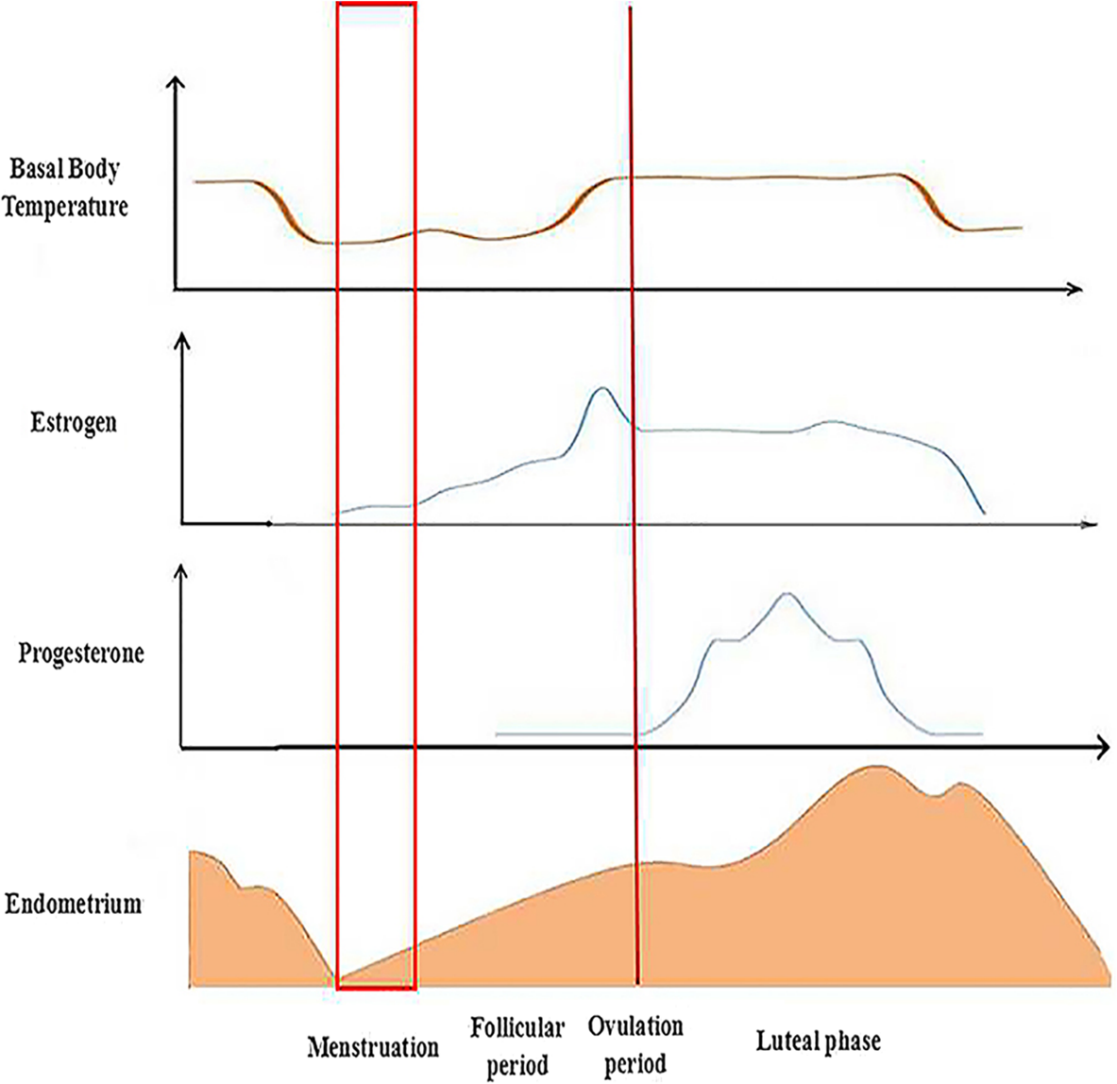
The changes of biology during different estrus cycle. Estrogen and progesterone are secreted via the ovary or uterus and undergo changes during the estrus cycle. In addition, the basal body temperature also changes, while the thickness of the endometrium has corresponding transformations. After menstruation, the new estrus cycle starts to develop. During the follicular period, the level of the basal body temperature and estrogen gradually rise. The thickness of the endometrium also increases. The levels of basal body temperature and estrogen maintain certain concentrations until the ovulation period. Thus, progesterone starts to increase. With the appearance of the luteal phase, all changes are restored until the end of menstruation

It is well known that the development of ovarian follicles is a continuous process (Fig. 1). There are five stages in female mammals. During these stages, the structure of the endometrium undergoes some changes. Estrogen and progesterone are secreted via the ovaries and uterus and undergo changes during the estrus cycle. In addition, the basal body temperature also changes, while the thickness of the endometrium undergoes the corresponding transformation [13] (Fig. 2).

**Fig. 3:**
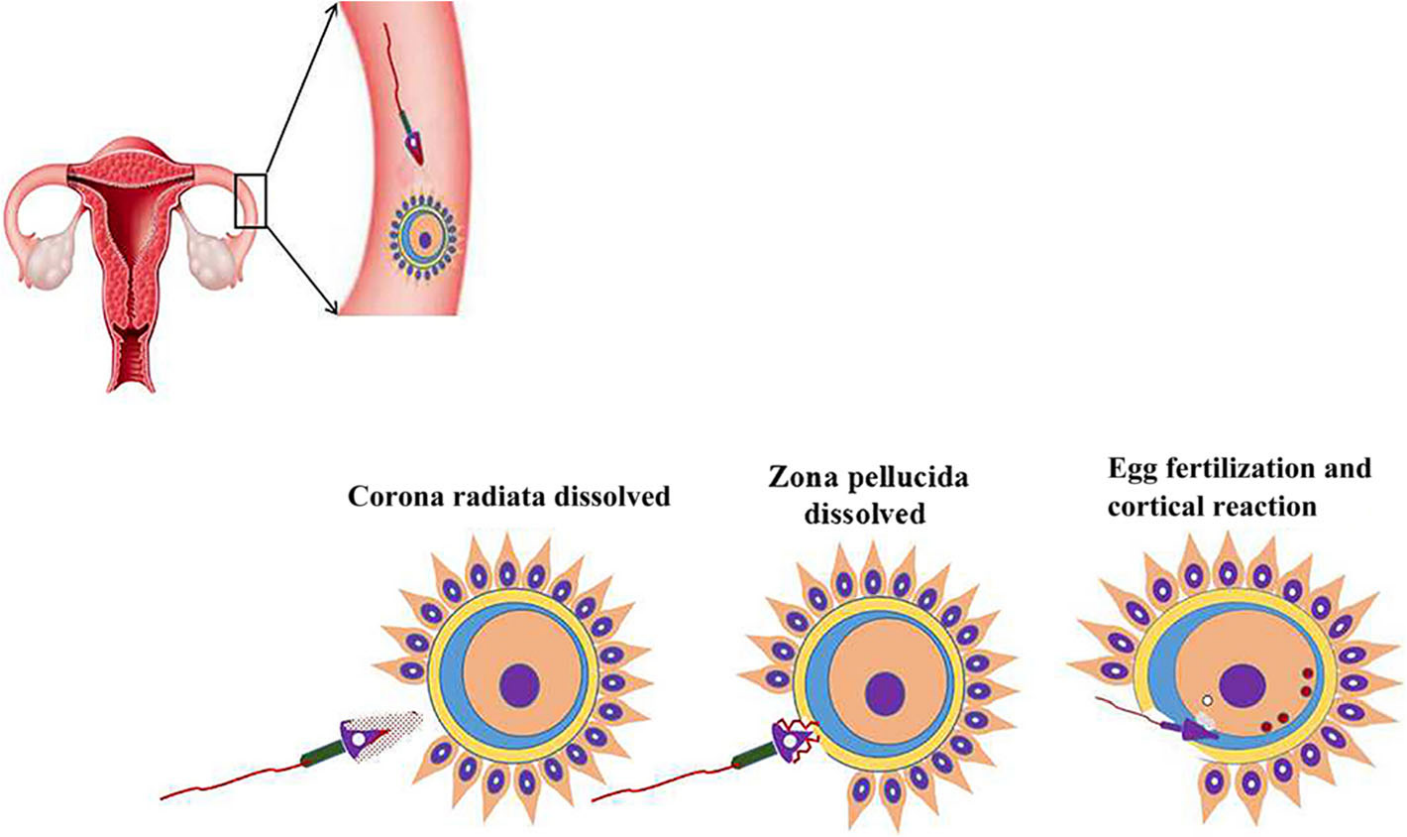
Fertilization processes of most viviparous and ovoviviparous animals. In most viviparous and ovoviviparous animals, the sperm and oocyte combine at the fallopian tube ampulla. In the picture, the first zygote shows a radiation crown dissolving; the second zygote shows the zona pellucida dissolving; the last zygote shows fertilized eggs and cortical response

**Fig. 4:**
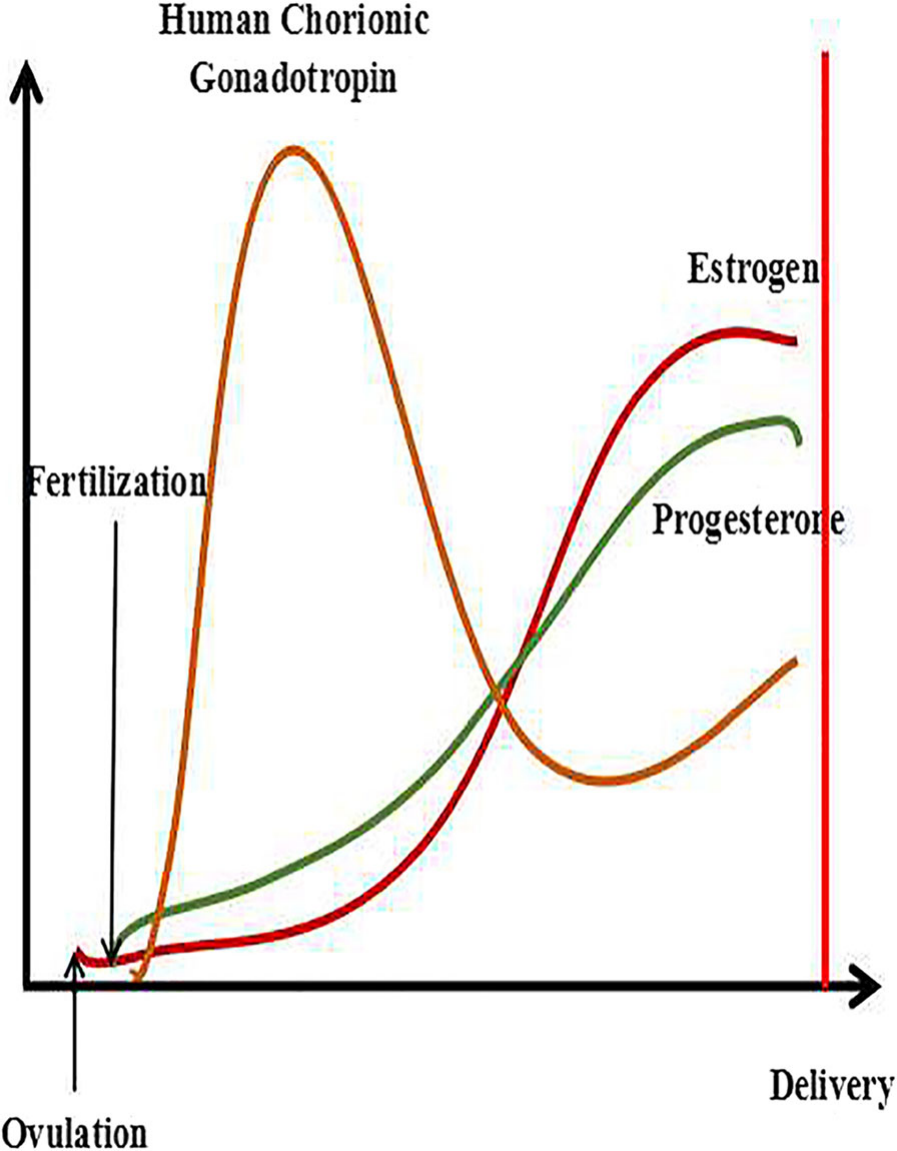
The trends of HCG, estrogen and progesterone during pregnancy. The yellow line represents the change in HCG. The green line represents the change in progesterone. The red line represents the level of estrogen. The final results of ovulation include two impacts, one of which is output in the body, called menstruation, and the other of which is combines with sperm, called fertilization. The level of hormones changes after fertilization; in particular, hCG immediately increases to the highest level. However, the levels of estrogen and progesterone slowly increase to stable concentrations, while hCG begins to drop to a certain extent

**Fig. 5:**
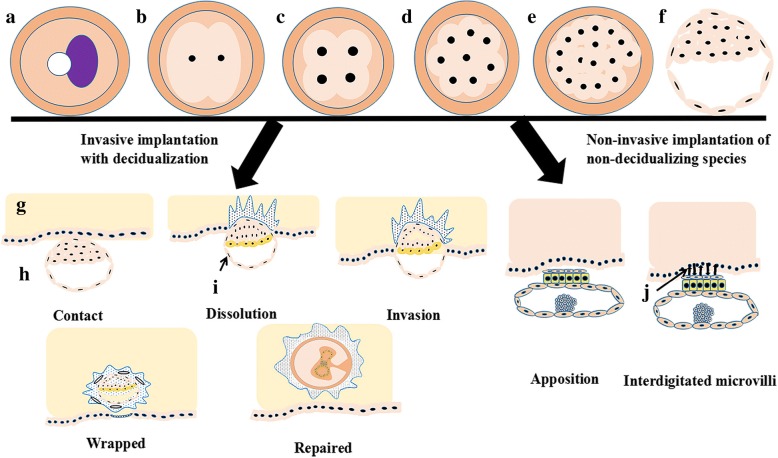
The process of implantation (including invasive implantation with decidualization and non-invasive implantation of non-decidualizing species) **a**: Zygote; **b**: 2 cells; **c**: 4 cells; **d**: 8 cells; **e**: Morula; **f**: Blastocysts; **g**: Endometrium; **h**: Uterine cavity; **i**: Trophoblast cells; **j**: Microvilli. Estradiol (E2) produced by the developing ovarian follicles interacts with progesterone produced by the CL to prepare the endometrium receptivity necessary for embryo implantation. The meeting of the oocyte and sperm and subsequent fertilization occur in the ampulla of the oviduct, followed by early embryo development within the oviduct, and the morula migrates to the uterus, where implantation occurs. The appearance of a fluid-filled inner cavity (blastocoel) is accompanied by cellular differentiation: the surface cells become the trophoblast and give rise to the extra-embryonic tissues, including the placenta, while the inner cell mass gives rise to the embryo and finally shedding of the zona pellucida, followed by orientation, apposition, attachment and adhesion of the blastocyst to the endometrium. If the blastocyst was not present, the CL would regress, and the uterus would start the cycle again. The time and chronological events of implantation differ among mammalian species irrespective of the length of gestation. In contrast to humans, horses, primates and rodents, in which implantation occurs shortly after the hatching of the blastocyst, the blastocyst in domestic ruminants and pigs elongates before implantation (the time to implantation: in pigs, the 14th day; in sheep, the 16th day; and in cattle, the 18th day), and this unique developmental event does not occur in the laboratory or in rodents or humans

The combination of sperm and egg consists of three steps: corona radiata dissolution; zona pellucida dissolution; and egg fertilization and cortical reaction (Fig. 3). The process occurs in the ampulla portion of the fallopian tube. Pregnancy starts when the fertilized egg is formed. Human chorionic gonadotropin (HCG) increases first and then decreases. Both estrogen and progesterone are increased during pregnancy (Fig. 4). It is noted that the process of implantation is significant in reproductive events. The process includes contact, dissolution, invasion, wrapping and repair (Fig. 5). Ovarian function and blastocyst development from ovulation to implantation are common in many mammalian species after ovulation (Fig. 5) [14].

## Oxidative stress

### Reactive oxygen species (ROS)

ROS are a double-edged sword: they not only play important roles as secondary messengers in many intracellular signaling cascades, but they also exert indispensable effects on pathological processes involving the generation of excessive ROS. The three major types of ROS are superoxide anion (O_2_^−^•), hydrogen peroxide (H_2_O_2_), and hydroxyl (•OH).

Most ROS are produced when electrons leak from the mitochondrial respiratory chain, also referred to as the electron transport chain (ETC) [15]. According to an estimate, up to 2% oxygen consumed can be diverted to the production of ROS formation by mitochondria, especially at complexes I and III [16]. The free radical superoxide anion (O_2_^−^•) is formed by the addition of one electron to ground state dioxygen, but it is unstable in aqueous solutions due to its being able to react spontaneously with itself, producing hydrogen peroxide (H_2_O_2_) and molecular oxygen (O_2_) (reaction 1). It can reduce Fe^3+^ to Fe^2+^and transform into O_2_ (reaction 2). H_2_O_2_ is not a free radical, but it is very harmful to cells because it is able to cross biological membranes and break down into the highly reactive hydroxyl radical (•OH).

The main source of hydroxyl radical is the metal-catalyzed Haver-Weiss reaction (reaction 3), the second of which is the Fenton-type reaction (reaction 4).

1 $$ {{\mathrm{O}}_2}^{-}\bullet +{{\mathrm{O}}_2}^{-}\bullet +2{\mathrm{H}}^{+}\to {\mathrm{H}}_2{\mathrm{O}}_2+{\mathrm{O}}_2 $$

2 $$ {{\mathrm{O}}_2}^{-}\bullet +{\mathrm{Fe}}^{3+}\to {\mathrm{O}}_2+{\mathrm{Fe}}^{2+} $$

3 $$ {{\mathrm{O}}_2}^{-}\bullet +{\mathrm{H}}_2{\mathrm{O}}_2\to {\mathrm{O}}_2+{\mathrm{O}\mathrm{H}}^{-}+\bullet \mathrm{OH}\ \left(\mathrm{Haver}-\mathrm{Weiss}\ \mathrm{reaction}\right) $$

4 $$ {\mathrm{Fe}}^{2+}+{\mathrm{H}}_2{\mathrm{O}}_2\to {\mathrm{Fe}}^{3+}+{\mathrm{O}\mathrm{H}}^{-}+\bullet \mathrm{OH}\ \left(\mathrm{Fenton}-\mathrm{type}\ \mathrm{reaction}\right) $$

## The defense mechanism against oxygen free radicals

### Primary defenses

**Fig. 6:**
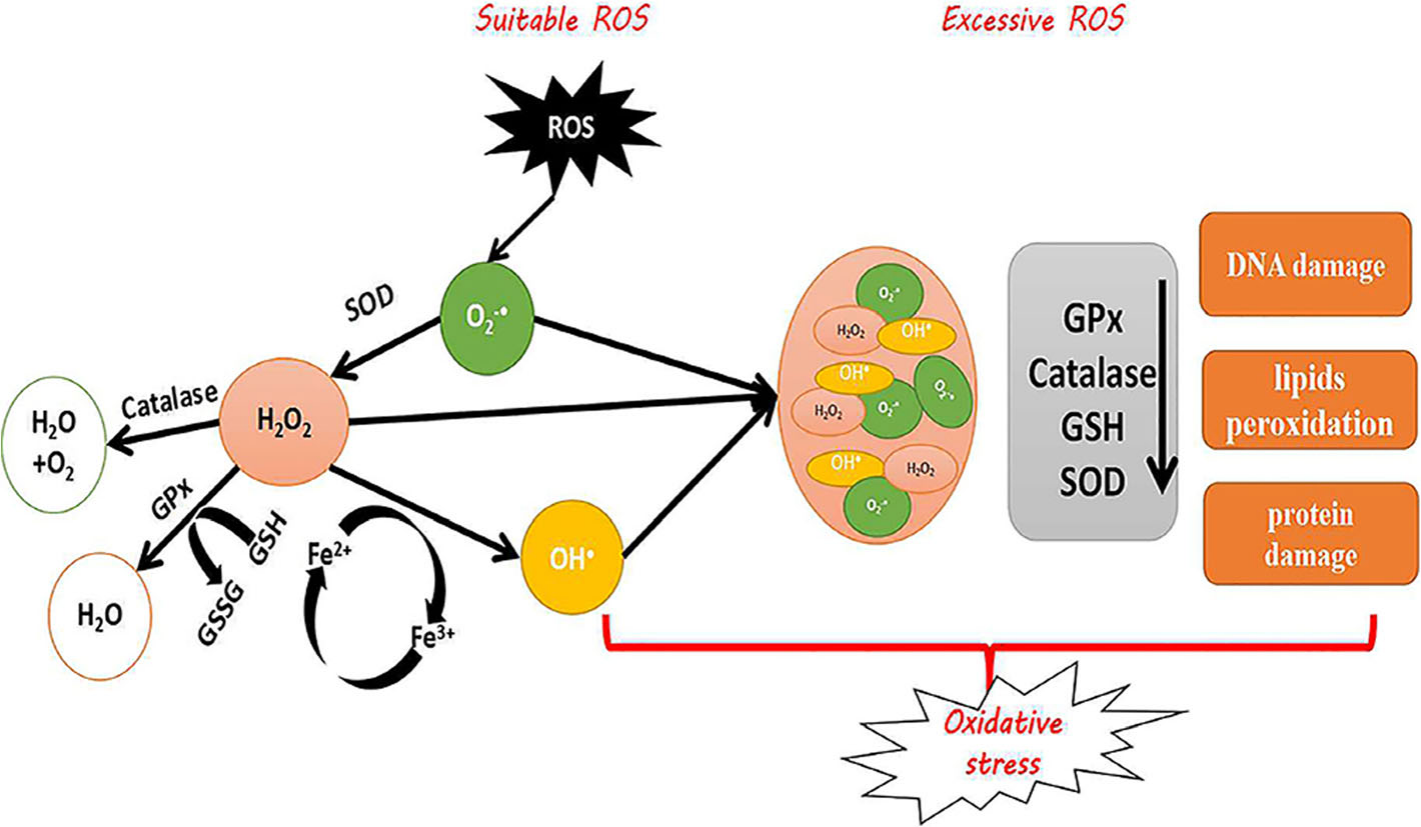
The defense mechanism against oxygen free radicals. SOD: Superoxide dismutase; GPx: Glutathione peroxidase; GSSG: Glutathione oxidase; GSH: Glutathione reductase; ROS: Reactive oxygen species; O_2_^−^•: Superoxide; H_2_O_2_: Hydrogen peroxide; •OH: Hydroxyl

As we all know, SOD, CAT, GPx and GSR belong to the primary defense mechanism (Fig. 6). SOD catalyzes O_2_^−^• dismutation to produce H_2_O_2_ and O_2_ at a rate 10^4^ times higher than spontaneous dismutation at the physiological pH [17, 18]. CAT is the enzyme that removes H_2_O_2_ from the cell when the latter is at high concentrations (reaction 5) [19]. GPx is an enzyme that catalyzes the reduction of H_2_O_2_ and organic free hydroperoxides requiring glutathione as a co-substrate (reaction 6 and 7) [20]. GSR is a cytosolic protein with a tissue distribution similar to that of GPx. The enzyme reduces oxidized glutathione, utilizing NADPH generated by various systems (reaction 8) [21].

5 $$ 2{\mathrm{H}}_2{\mathrm{O}}_2\to 2{\mathrm{H}}_2\mathrm{O}+{\mathrm{O}}_2 $$

6 $$ {\mathrm{H}}_2{\mathrm{O}}_2+2\mathrm{GSH}\to \mathrm{GSSG}+2{\mathrm{H}}_2\mathrm{O} $$

7 $$ \mathrm{ROOH}+2\mathrm{GSH}\to \mathrm{GSSG}+\mathrm{ROH}+{\mathrm{H}}_2\mathrm{O} $$

8 $$ \mathrm{GSSG}+\mathrm{NADPH}+{\mathrm{H}}^{+}\to 2\mathrm{GSH}+{\mathrm{NADP}}^{+} $$

### Secondary defense

The existence has been reported of an enzyme with peroxidase activity called phospholipid hydroperoxidase GPx, which is capable of reducing lipid hydroperoxides without the action of phospholipase A2 [22]. In addition, different oxidoreductases that catalyze reduction reactions of thiol and other protein groups when these molecules are oxidatively damaged are protective enzymes against oxygen free radicals. Nuclear enzymes for DNA repair are considered to be defense systems against oxidative injury by oxygen free radicals [23]. Vitamin E, the major lipid-soluble antioxidant present in all cellular membranes, protects against lipid peroxidation. The tocopheryl radical might also be directly reduced by the ascorbic acid-GSH redox couple. β-carotene exerts the most efficient scavenger action with vitamin E, while β-carotene acts at low oxygen pressures. However, vitamin E protects conjugated double bonds of β-carotene from oxidation.

In all, OS plays an important role in the pathophysiology of complicated pregnancies. OS was described as an imbalance in the generation of ROS [1]. These ROS are oxygen free radicals produced by the reduction in molecular oxygen and generated as byproducts of aerobic respiration and metabolism. These molecules are capable of activating and modulating various signaling pathways, including those involved in cell growth, differentiation and metabolism [24]. They can also induce cellular oxidative damage by interacting with DNA and intracellular macromolecules, such as protein and membrane lipids, so they can lead to cellular malfunction that can initiate pathological processes.

## Oxidative stress in ovary

ROS affect a variety of physiologic functions of the ovary, including ovarian steroid genesis, oocyte maturation, ovulation, formation of blastocysts, implantation, luteolysis and luteal maintenance in pregnancy. OS is an important modulator of ovarian germ cell and stromal cell physiology [25]. Concentrations of ROS could also play a major role in the implantation and fertilization of eggs, and a relevant study showed localization of SOD in the ovary and found that copper-zinc SOD (Cu-Zn SOD) was localized in the granulose cell of growing follicles and mature Graafian follicles, as well as manganese superoxide dismutase (MnSOD) being localized in luteal cells of the corpus luteum in rats [26].

ROS exert both negative and positive effects on mammalian ovaries [27]. ROS affect multiple physiological and pathological activities in the ovaries, from oocyte maturation to fertilization. In cycling ovaries, different markers of OS are negatively affected [28, 29]. Macrophages, leukocytes, and cytokines present in the follicular fluid microenvironment are major sources of ROS. ROS in the follicular fluid join in follicular growth, oocyte maturation, and ovarian steroid biosynthesis [15]. At the same time, a critical process for ovarian folliculogenesis, dominant follicle selection, CL formation and embryo formation is angiogenesis [30, 31], which is a complex process. It is promoted by estrogens that regulates some cellular factors, such as VEGF [32]**.** ROS produced from NADP(H) oxidase were shown to be significant for angiogenesis in vivo and VEGF signaling in vitro [33]. Accordingly, ROS are involved in follicular growth in part by regulating angiogenesis.

The appropriate amount of ROS is required for ovulation. ROS produced by the preovulatory follicle are considered critical inducers of ovulation**,** and inhibition of ROS has been confirmed to disturb ovulation [27, 34]. Oxygen deprivation stimulates follicular angiogenesis, which is important for abundant growth and development of ovarian follicles [35]. The development of follicles from the primordial stage to antral follicles is accompanied by a marked increase in the metabolic function of granulosa cells, especially a large increase in cytochrome P450 activity with steroid biosynthesis [36]. Large amounts of ROS are produced during electron transport, indicating that functional granulosa cells are related to the pro-oxidant state in the follicles. ROS are induced in preovulatory follicles with oscillation of prostaglandins, cytokines, proteolytic enzymes, and steroids, resulting in blood flow alterations and eventual follicle rupture [37]. With the exception of dominant follicles, which are released for fertilization, the other growing follicles all undergo apoptosis, and this process is promoted by ROS. In parallel, follicle-stimulating hormone (FSH)-induced estrogen synthesis and upregulation of CAT and GSH in growing follicles resist the apoptotic process to maintain the balance during normal ovarian function [38]. ROS are generated in the CL and are involved in functional luteolysis. ROS and antioxidants are related to progesterone synthesis in the luteal phase [35] (Table 1).

**Table 1:** The role of oxidative stress in the female reproductive process

Function	Reproductive process	Reference
Positive effect	Zn-Cu SOD↑ → Promotion of the development of follicles	[26]
	Biosynthesis of ovarian steroids → P450↑ → ROS↑ → Blood flow↑ → Rupture of follicles → Ovulation	[36, 37, 40, 41]
	ROS↑ → Promotion apoptosis of non-dominant follicles FSH↑ → E2↑ → CAT and GSH↑ → Protection of cells from apoptosis	[38]
	E2 and P ↓ → SOD↓ → OS↑ → Endometrial shedding and implant failure	[62]
	ROS↑ → NF-κB↑ → PGF_2α_↑ → Luteum dissolution	[35, 39, 61]
	Sperm-ovum binding → ROS↑ → Corpus luteus functional↑ ROS↑ → Antioxidants↑ → Synthesis of progesterone	[39]
Negative effect	PCOS: Serum proline activity↑, OS↑ Physiological hyperglycemia → ROS↑ (Monocytes) → TNF-α↑ → NF-κB↑ → Resistance of Insulin↑	[45, 46]
	Preeclampsia: Defective placenta → Hypoxia and reperfusion injury → OS↑ → Cytokines↑, Prostaglandins↑ → TAS↓, GPx of placenta↓ V_C_↓ → Risk of preeclampsia↑ (MDA↑) ROS↑ → Vasoconstriction↑ → Coagulation activity↓ OS↑ → Vascular endothelial injury↑ → ROS↑ → TNF-α↑, ox-LDL↑ → Endothelial subtypes of activated NAD(P)H oxidase → SO anion↑ Auto-antibodies of AT1-AA↑ → NAD(P)H oxidase↑ → ROS↑ → SO anion↑	[67, 76–81, 83–85]
	Endometriosis: In the peritoneal fluid, MDA↑, IL-6↑,TNF-α↑, IL-8↑, VEGF↑, MCP-1↑, ox-LDL↑. In endometriotic lesions: OS↑ → NF-κB↑ → Inflammation↑ In endometriotic cells: MAPK↑ ROS↑ → IUGR, abortion, fetal malformation	[71–73, 75]

The ‘→’ indicates that it has an effect on the next step. The ‘↑’ represents an increase and the ‘↓‘represents a decrease

However, excessive deprivation of oxygen will also cause some damage to follicles, as we discuss in the following. Cu/Zn-SOD is increased in the CL during the early to mid-luteal phase, but it is decreased during the regression phase, which could explain the increase in ROS concentrations during regression, and this change in activity is similar to that in progesterone concentrations. Other possible explanation for the decrease in Cu-Zn SOD during the regression phase is an increase in prostaglandin PGF_2α_ or macrophages; another reliable explanation is a decrease in ovarian blood flow [35]. Prostaglandin F_2α_ stimulates production of the SO anion by luteal cells and phagocytic leukocytes in the CL. The reduction of ovarian blood flow causes tissue damage by ROS production. However, the concentration of Mn-SOD in the CL during regression is increased, thus scavenging the ROS produced in the mitochondria by inflammatory reactions and cytokines. Therefore, complete disruption of the CL leads to a significant decrease in Mn-SOD in the regressed cells, and cell death is imminent [39]. In addition, ROS also participate in mammalian ovulation and follicular rupture. The generation of the two processes is the result of vascular changes or the proteolytic cascade. The crosstalk between these two cascades is mediated by ROS, cytokines, and vascular endothelial growth factor (VEGF) [40, 41]. Ad4BP, a zinc finger DNA-binding protein, was identified as a transcription factor regulating steroidogenic P-450 genes in a cAMP-dependent manner [42], and a recent study showed that the correlation between Ad4BP and SOD expression suggested an association between OS and ovarian steroid genesis. Both human granulosa and luteal cells respond to hydrogen peroxide with extirpation of gonadotropin action and inhibition of progesterone secretion. Hydrogen peroxide lowers both cAMP-dependent and non-cAMP-dependent steroidogenesis [28, 43]. As we all know, OS influences the entire reproductive process of women’s lives. ROS attacks the 8th carbon atom of guanine in DNA to generate 8-hydroxy-deoxyguanosine (8-OHDG), which is an oxidized derivative of deoxyguanosine, the levels of which are higher in aging oocytes [44]**.** 8-OHdG is the most familiar base modification in mutagenic damage. 8-OHdG causes base mutation and mismatches in DNA replication, resulting in G mutations to T and G:C to T:A transversion. Therefore, 8-OHdG has become a marker for OS.

OS has been implicated in different female diseases, including PCOS, which is the most common endocrine abnormality of reproductive-aged women and has a prevalence of approximately 18%. It is a disorder characterized by hyperandrogenism, ovulatory dysfunction, and polycystic ovaries [45]. Various studies have reflected the presence of OS in PCOS patients. In a study by Hilali et al., PCOS patients had increased serum prolidase activity, as well as higher total oxidant status and OS indices—the ratio of oxidants to total antioxidant status. The decrease in mitochondrial O_2_ consumption and GSH levels, along with increased ROS production, explains the mitochondrial dysfunction in PCOS patients. Physiological hyperglycemia generates increased levels of ROS from mononuclear cells, which activate the release of tumor necrosis factor-α (TNF-α) and increase the inflammatory transcription factor nuclear factor-kappa B (NF-κB). As a result, concentrations of TNF-α, a known mediator of insulin resistance, are further increased. The resultant OS creates an inflammatory environment that further increases insulin resistance [46], causing abnormal ovarian extracellular remodeling, multiple cyst formation, and chronic anovulation, leading to infertility [47] (Table 1).

Recently, PCOS has been paid significant attention because it exerts severe effects on female reproduction. In this review, we collect some interesting evidence that implies a delicate relationship between stress and PCOS [48]. Its etio-pathogenesis and pathophysiology include roles for genetic, environmental and endocrine factors. Franks et al. defined PCOS as a gene-dependent ovarian pathology, characterized by the overproduction of androgens and not uniformly represented by the interaction of genetic “propensities” with other genetic and environmental factors [49]. Thus, PCOS seems to be a genetic disease, but after investigation by Escobar-Morreale HF et al. found that it is caused by the interaction of susceptibility and protective genetic variants, and these mutations could be chosen due to survival advantages in the evolution process, requiring an accurate study of environmental factors, including race, diet and lifestyle [50]. V. De Leo et al. reported that disruption of the delicate balance between intra-ovarian and extra-ovarian factors alters and impairs the formation of mature oocytes, leading to infertility. In addition, insulin plays a particular role in PCOS, and in vitro studies have demonstrated that insulin stimulates thecal cell proliferation, increases secretion of androgens mediated by LH and increases cytochrome P450 expression of LH and IGF-1 receptor [51]. As we all know, P450 can increase ROS. The above evidence indicates that ROS join in the pathological process of PCOS.

## Oxidative stress in the uterus and placenta

Pregnancy itself is a state of OS, arising from the increased metabolic activity in the placental mitochondria and increased ROS production due to the higher metabolic demand of the growing fetus [52, 53]. Superoxide (SO) anions produced by the placental mitochondria appear to be a major source of ROS and lipid per-oxidation contributing to OS in the placenta [54]**,** supported by mitochondrial production of lipid peroxides, free radicals, and vitamin E in the placenta, which increases as gestation progresses[55]**.** In the second trimester, the placenta gradually matures and increases in size, with less hairiness and wider blood vessels. The cytotrophoblast becomes a single cell and gradually replaces the endothelial layer covering the smooth muscle of the spiral artery. Slowly, maternal blood penetrates from the mother’s spiral artery into the interstitial space [55–57]. During this process, placental tissue forms a large amount of free radicals, and oxidation occurs. Intense stress results, but the placenta gradually adapts to this environment and returns to normal under the action of antioxidant activity [58, 59]. While physiological concentrations of endogenous glucocorticoids are supportive of fetal development, excessive glucocorticoids in utero (i.e., maternal stress) adversely affect mammalian offspring by “programming” abnormalities that are primarily manifested postpartum [60]. ROS are also believed to play a role in the different phases of the endometrial cycle. The late luteal phase is characterized by elevated levels of lipid peroxide and a decrease in the antioxidant SOD. ROS stimulate the secretion of PGF_2α_ through activation of NF-κB [61]. Decreased levels of estrogen and progesterone lead to decreased SOD expression and hence generate OS in the uterus, resulting ultimately in endometrial shedding and lack of implantation. Controlled levels of ROS have, however, been associated with angiogenic activity in the endometrium, causing regeneration during every cycle. These studies showed that limited levels of ROS are necessary to maintain physiological function, but when present in higher concentrations, ROS can have deleterious effects [62]. In other words, although a physiological balance between ROS and antioxidant activity is maintained in normal pregnancies [63], an imbalance can increase OS. The placenta experiences a heightened level of OS in certain pathologic conditions of pregnancies, including gestational diabetes, fetal growth restriction, preeclampsia and miscarriage [64–66].

OS leads to endothelial cell dysfunction. In the uterus, endothelial cell dysfunction results in many diseases, such as preeclampsia and endometriosis. There are many causes that induced endothelial cell dysfunction. TNF-α, a plasma cytokine, has been demonstrated to cause endothelial cell injury, but the antioxidant Mn-SOD neutralizes SO anions generated by the cytokine TNF-α. This process is a self-protective mechanism against TNF-α-induced OS. In addition, defective placentation leads to placental hypoxia and reperfusion injury due to ischemia, and the resultant OS triggers the release of cytokines and prostaglandins, resulting in endothelial cell dysfunction and playing an important role in the development of preeclampsia [67]. In addition, ROS generated from NADP (H) oxidase are critical for VEGF signaling in vitro and angiogenesis in vivo [33]. Small amounts of ROS are produced from endothelial NADP (H) oxidase activated by growth factors and cytokines. ROS generated in and around the vascular endothelium could play a role in normal cellular signaling mechanisms. They might also be important causative factors in endothelial dysfunction.

Endometriosis is a benign, estrogen-dependent, chronic gynecological disorder characterized by the presence of endometrial tissue outside the uterus. There was a report suggested that the elevated ROS causing OS are produced by erythrocytes and apoptotic endometrioma cells, as well as the activated macrophages that are recruited to phagocytize apoptotic cells [10]. Additionally, the ROS producing enzyme xanthine oxidase, which is considered another contributor to excess ROS, are expressed in greater quantities in women with endometriosis [68]. OS plays a large role in infertility. Another way in which cells are damaged through OS is via lipid peroxidation, which is the oxidative destruction of polyunsaturated fatty acids in the plasma membrane [69]. This leads to “increased membrane permeability, degraded membrane integrity, inactivated enzymes and structural damage of the DNA; cell death rapidly follows” [70]**.** In addition, OS induces local inflammation, resulting in elevated levels of cytokines and other factors that promote endometriosis, as discussed later [8].

The peritoneal fluid of patients has been found to contain high concentrations of malondialdehyde (MDA), pro-inflammatory cytokines (IL-6, TNF-α, and IL-1β), angiogenic factors (IL-8 and VEGF), monocyte chemoattractant protein-1 (MCP-1) [71], and oxidized LDL (ox-LDL). Pro-inflammatory and chemotactic cytokines play central roles in the recruitment and activation of phagocytic cells, which are the main producers of ROS and RNS. Activation of NF-κB by OS has been detected in the endometriotic lesions and peritoneal macrophages of patients with endometriosis [72]. Signaling mediated by NF-ĸB stimulates inflammation, invasion, angiogenesis, and cell proliferation, and it also promotes the apoptosis of endometriotic cells. Additionally, N-acetylcysteine (NAC) and vitamin E are antioxidants that limit the proliferation of endometriotic cells, likely by inhibiting activation of NF-κB [73]. A study indicated a therapeutic effect of NAC and vitamin E supplementation on endometriotic growth [74]. Similar to tumor cells, increased ROS and subsequent cellular proliferation in endometriotic cells activate of mitogen-activated protein kinase (MAPK) and extracellular regulated kinase (ERK1/2) [75]. More seriously, the increase in ROS in endometriosis patients can lead to adverse effects on embryos, such as IUGR, spontaneous abortion, or fetal dysmorphogenesis [69] (Table 1).

Preeclampsia is a vascular pregnancy disorder that often involves impaired placental development. It is a complex multisystem disorder that can affect normotensive women. It can cause the poor implantation and growth restriction observed in preeclampsia because OS causes increased nitration of p38 MAPK, resulting in a reduction in its catalytic activity.

Increased ROS concentrations in patients with preeclampsia have been proved by the increased levels of MDA, an index of lipid peroxidation [76]. Under normal conditions, the impairment of circulatory homeostasis is caused chiefly by vascular endothelial dysfunction in preeclampsia. It is characterized by the tendency to cause vasoconstriction and low anticoagulant activity. ROS seem to play a critical role in the endothelial dysfunction associated with preeclampsia [77]. In other words, the pathologic event in preeclampsia is injury to the vascular endothelium regulated by OS from increased placental ROS [78] or decreased antioxidant activity [79].

There are many reasons for the increase in ROS. For instance, neutrophil modulation occurring in preeclampsia is an important source of ROS, resulting in increased production of the SO anion and decreased levels of NO, ultimately causing endothelial cell damage in patients with preeclampsia. Levels of TNF-α and oxLDL are increased in preeclampsia and have been shown to activate the endothelial isoform of NAD(P)H oxidase, ultimately resulting in increased levels of the SO anion. These results suggest that the consumption of antioxidants to counteract heightened lipid per-oxidation might injure the vascular endothelium and could be involved in the pathogenesis of preeclampsia [80].

In addition, autoantibodies against the angiotensin receptor AT1, particularly the second loop (AT1-AA), can stimulate NAD(P)H oxidase, leading to increased generation of ROS [81]. The AT1 receptor of preeclamptic women has been observed to promote both the generation of the SO anion and over-expression of NAD(P)H oxidase in cultured trophoblasts and smooth muscle cells. Therefore, early placental development can be affected by dysregulated vascular development and function secondary to NAD(P)H oxidase-mediated altered gene expression [82]. Preeclamptic women produce ROS and exhibit higher NAD(P)H expression than those without the disease [35]. More specifically, it has been reported that women with early-onset preeclampsia produce larger amounts of the SO anion than women with late-onset disease [83]. Affected women also have decreased total antioxidant status (TAS) and placental GPx [84] and low levels of vitamins C and E. Lack of vitamin C intake seems to be associated with an increased risk of preeclampsia, and some studies have shown that peri-conceptional supplementation with multivitamins can lower the risk of preeclampsia in normal or underweight women [85].

There have been studies focusing on the effects of restraint stress on uterine and embryo implantation in pregnant mice. In these studies, uterine local micro-environment changes and uterine histomorphology research were emphasized. Liu Guanhui et al. reported that the mice were subjected to restraint stress from embryonic day1 (E1). This study demonstrated that restraint stress increased the level of corticosterone (CORT) in plasma, and uterine natural killer (uNK) cells in the endometrium were significantly increased, accompanied by the decreased density of mast cells in the myometrium. In addition, restraint stress markedly decreased the CD3^+^CD4^+^ T/CD3^+^CD8^+^ T cell ratio. Additionally, antioxidant ability was compromised, and the concentration of MDA was increased [86]. Moreover, restraint stress reduced the weight of the uterus and ovary and the intake of food with reduction in weight, while the relative endometrial area and uterine gland area were reduced after restraint stress. In addition, restraint stress decreased micro-vessel density and VEGF expression [87].

## The signaling molecules between oxidative stress and reproduction

OS has led to a variety of signaling pathways, resulting in crosstalk among many protein factors in the body. Especially in the female reproductive organs, OS leads to a series of abnormal events in egg production and ovulation. During pregnancy, implantation will be impaired, leading to loss of embryos and changes in local immune function in the uterus. Research on these signals is currently the most important concept in this field and is of great significance to the reproduction of female animals.

Before this review, there were a number of reviews discussing the contact between reproduction and OS. For example, Perucci et al. proposed a hypothesis that the ADAMs pathway protects women from the inflammatory lesions of preeclampsia [88]. Wu et al. elaborated on potential therapeutic approaches to placental stress by exploring the relationship between OS and apoptosis and between OS and cellular autophagy, resulting in speculation about a comprehensive therapeutic target [89]. Sultana et al. fully summarized the adverse pregnancy outcomes caused by aging placentas, explaining the mechanisms of telomerase and placental disorders [59]. Wojsiat et al. explained the effects of OS on oocyte and fertilization outcomes and the effect of overproduction of active substances on in vitro fertilization [90]. Nevertheless, our review not only summarizes the above discussion but also makes reasonable assumptions about the signaling pathways in reproductive diseases.

Hypoxia and inflammation lead to the production of TNF-α, which induces the release of large amounts of ROS from the mitochondria in cells. Excessive ROS cause an imbalance between oxidation and antioxidation, leading to OS. The body’s signaling pathway will evince a series of changes following to exposure to the dual impact of OS and inflammation. This article focuses on the collection of OS-induced reproductive disease-related signaling pathways, including the p38 MAPK pathway, the Kelch-like ECH-associated protein 1 (Keap1)-Nuclear factor erythroid 2-related factor 2 (Nrf2) pathway, the Jun N-terminal kinase (JNK) pathway, the forkhead transcription factors of the O class (FOXO) family, and apoptosis.

Nrf2 is a key molecule activated in response to OS, and it regulates antioxidant response to protect cell function [91]. Normally, Nrf2 binds to Keap1, is sequestered in the cytoplasm, and then is degraded by a proteasome pathway [92]. After activation, it transfers to the nucleus to activate a large number of antioxidant genes [93]. In other words, transcriptional activation of antioxidant defense genes and restoration of vascular redox homeostasis are necessary when OS occurs. Importantly, the redox-sensitive Keap1-Nrf2 pathway plays a key role in the process [94]. These studies also implied that Nrf2 deficiency caused fetal DNA damage and neurological deficits, and inactivation of Nrf2 has also been shown to underlie inflammation-induced trophoblastic apoptosis. As studies have progressed, the literature has increasingly revealed that Nrf2 plays a significant role in pregnancy and has highlighted the important role of Nrf2 in protecting the fetus in utero OS [95]. Nrf2 is sensitive to maternal immunological status. In normal pregnancy, Nrf2 is only decreased after term vaginal delivery. However, notably, the expression of Nrf2 is significantly reduced when the uterus is infected [96]. Furthermore, the mechanism of Nrf2 antioxidant defense plays an important role in adverse pregnancy priming. Nrf2 is a regulator of antioxidant defense in vascular dysfunction and oxidative damage [95]. Many studies have shown that suitable OS increased Nrf2 and the expression of downstream targets, such as heme oxygenase 1 (HO-1), NAD(P)H: quinoneoxidoreductase (NQO1), and glutamate-cysteine ligase subunit catalysis (GCLC), etc., to resist OS [97]. However, as described above, we speculate that the activity of Nrf2 significantly decreases, and Keap1 binds to Nrf2 more strongly when excessive OS causes severe inflammation in the uterus. Related studies have shown that FOXO3 participates in the interaction between Keap1 and Nrf2. Loss of FOXO3 leads to severe inactivation of Keap1, which in turn cannot prevent the activation of Nrf2, which is a very important finding in tumors. Research also revealed the important role of FOXO3 in the Keap1-Nrf2 axis. At the same time, it is not denied that, in the absence of FOXO3, Nrf2 is activated under the induction of AKT and protects cells from damage due to OS by this form [98]. Therefore, we hypothesize that, if OS induces inflammation in the reproductive system, the changes in FOXO3 affect the interaction between Keap1 and Nrf2, which could be a marker of damaging OS in our study (Table 2).

**Table 2:** The important proteins in reproductive mechanisms

Protein	Function and role in reproductive process	Interaction between proteins	Reference
Keap1-Nrf2 pathway	Keap1: Keap1 binds to Nrf2 in cytoplasm. Nrf2: Protects cells under moderate oxidative stress A regulator of antioxidant defense in vascular dysfunction and oxidative damage. Deletion of Nrf2 → Fetal DNA damage and nervous system defects (the basis of trophoblast cell apoptosis). Vaginal delivery and Uterine infection: Nrf2↓	The right amount of OS → Nrf2↑ → HO-1↑,NQO1↑,GCLC↑ Excessive OS → FOXO3↑ → Ability of Keap1 to bind to Nrf2↑ Deletion of FOXO3: OS↑ → AKT↑ → Nrf2↑	[91–93, 95–98]
NF-κB pathway	An active molecule in the immune system; Redox-Sensitive transcription factors Placental stress: OS → NF-κB↑ → Pro-inflammatory Cytokines↑ → Placental apoptotic process is activated Endometriosis: OS↑ → TNF-α↑ → NF-κB↑ In vitro: IL-1β → NF-κB↑ → MIF↑, TNF-α↑	Inhibitory IκB protein family binds to NF-κB in cytoplasm IKKB protein is degraded (IKKα, IKKβ, NEMO mediate) → NF-κB enters nuclear to regulate target gene. IKKβ↑ → p-FOXO3↑ Deletion of FOXO3 → NF-κB↓ FOXO3↑ → BCL10↑ → IKKB↓ → NF-κB↑ → Anti-apoptosis gene↑	[2, 99–104, 108, 109]
FOXO family	FOXO1(all tissues): Deletion of FOXO1, embryonic cell death due to incomplete blood vessel development FOXO3(all tissues): Deletion of FOXO1, lymphocyte proliferation, extensive organ inflammation FOXO4 (muscle, kidney, colorectal): Deletion of FOXO4, inflammation of the colon in response to inflammatory stimuli FOXO6(brain, liver): Deletion of FOXO6, shows normal learning, but memory consolidation is impaired FOXO1↑ → Apoptotic pathway in decidual stromal cells, and Inhibits endometrial epithelial cell growth	FOXO1↑ → WNT4↑,PRL↑,IGFBP1↑ JNK↑ → FOXO1↑ → MnSOD↑, CAT↑ → Protecting cells	[111, 112, 115–120]
MAPK family	JNK: Activated by stress and inflammation P38 MAPK: Activated by stress and inflammation ERK: Activated by inflammation and growth factors OS↑ → p38 MAPK↑ → Aging and premature aging of fetal tissue Endometriosis: ERK↑ Endometrial stromal cells: time of p-ERK↑; OS↑ → ERK↑,H_2_O_2_↑ → p-ERK↑	JNK↑ → FOXO1↑ → MnSOD↑, CAT↑ → Protecting cells P450↑ → ROS↑ → ASK1-P-p38 MAPK	[118, 121, 123–126, 129, 130]

The ‘→’ indicates that it has an effect on the next step. The ‘↑’ represents an increase and the ‘↓‘represents a decrease

NF-κB is an active molecule in the immune system. In mammals, the NF-κB family is composed of five related transcription factors: c-Rel, p50, p52, RelB and RelA (a.k.a. p65) [99]. NF-κB is the nodal point of a primary inflammation-stimulated signaling pathway that plays a significant role in the immune response [100], while NF-κB is also a redox-sensitive transcription factor [101]. Therefore, its effect is self-evident in OS, including embryonic stresses. The NF-κB pathway is activated when embryonic stresses occurs, and a variety of pro-inflammatory cytokines is increased. Then, the apoptotic process of the placenta is activated [2]. Therefore, this study indicated that NF-κB controls cell survival through the enhancement of anti-apoptotic gene transcription. In most cells, the NF-κB complex is inactive, and it is mainly present in the cytoplasm by binding to the inhibitory IκB protein family. When the NF-κB pathway is activated, the IκB protein is degraded, NF-κB complex enters the nucleus to modulate the expression of target genes, and the degradation of IκB protein is mediated through the IκB kinase (IKK) complex, which consists of two catalytically active kinases (IKKα and IKKβ) and the regulatory scaffold protein NEMO. In the activation pathway, IKKβ and NEMO are very necessary for activation of the complex [102]**,** while IKKβ also acts on other factors, such as Forkhead box O3, a transcription regulator. A study showed that FOXO3 is subject to IKKβ-mediated phosphorylation, leading to the nuclear exclusion and degradation of FOXO3 [103]. In addition, a study reported that FOXO3 was a positive regulator of NF-κB signaling and found that over-expression of FOXO3 increased and knockdown of FOXO3 repressed NF-κB activities. The study indicated that FOXO3 activated NF-κB by inducing expression of B-cell lymphoma/leukemia 10 (BCL10), an upstream regulator of inhibitor of kappa B kinase (IKK)/NF-κB signaling [104]. In reproductive stress diseases, for example, endometriosis, increased expression of NF-κB has been confirmed in cultured endometriotic stromal cells [105] and peritoneal macrophages isolated from women with endometriomas [106]. In any case, changes in NF-κB are strongly associated with inflammation. Endometriosis is a disease caused by OS during reproductive. OS leads to an increase in TNF-α, which in turn causes inflammation, and the NF-κB pathway is activated. Additionally, in vitro evidence raised the possibility that the changes might be due to the endometriotic microenvironment. IL-1β stimulates NF-κB with subsequent increased production of inflammatory cytokines [107], including macrophage migration inhibitory factor (MIF) in endometrial stromal cells [108] and TNF-α in the immortalized epithelial (12Z) cell line [109]. In conclusion, the NF-κB pathway is activated when reproductive OS occurs (Table 2).

FOXO1, the same family as FOXO3, is also involved in the processes of OS and pregnancy. The FOXO subfamily of Forkhead transcription factors is a direct downstream target of the PI3K/Akt pathway [110]. The mammalian forkhead transcription factors of the O class (FOXOs) number four: FOXO1, FOXO3, FOXO4, and FOXO6. Further, FOXO1 and FOXO3 exist in nearly all tissues. FOXO4 is highly expressed in the muscle and kidneys, and FOXO6 is primarily expressed in the brain and liver. They are involved in the processes of proliferation, apoptosis, autophagy, metabolism, inflammation, differentiation and stress tolerance [111]. However, FOXO1 plays a significant role in reproduction. It regulates cyclic differentiation and apoptosis in the normal endometrium [112]. Additionally, genome-wide expression profiling demonstrated that FOXO1 knockdown perturbs the expression of more than 500 types of genes in decidualizing human endometrial stromal cells [113]. In the past, many studies of human endometrium provided reliable evidence for this ability of FOXO transcription factors to regulate diverse genes in response to change hormones [114]. However, the interaction between progesterone and FOXO1 is even more striking. It is well known that progesterone exerts inhibitory effects on endometrial epithelial growth, and a study revealed this mechanism and showed that siRNA inhibition of FOXO1 significantly attenuated the effects of progestin in inhibiting endometrial epithelial cell growth. Therefore, FOXO1 is essential for the anti-proliferative effects of progesterone on both endometrial stromal and epithelial cells [115].

**Fig. 7:**
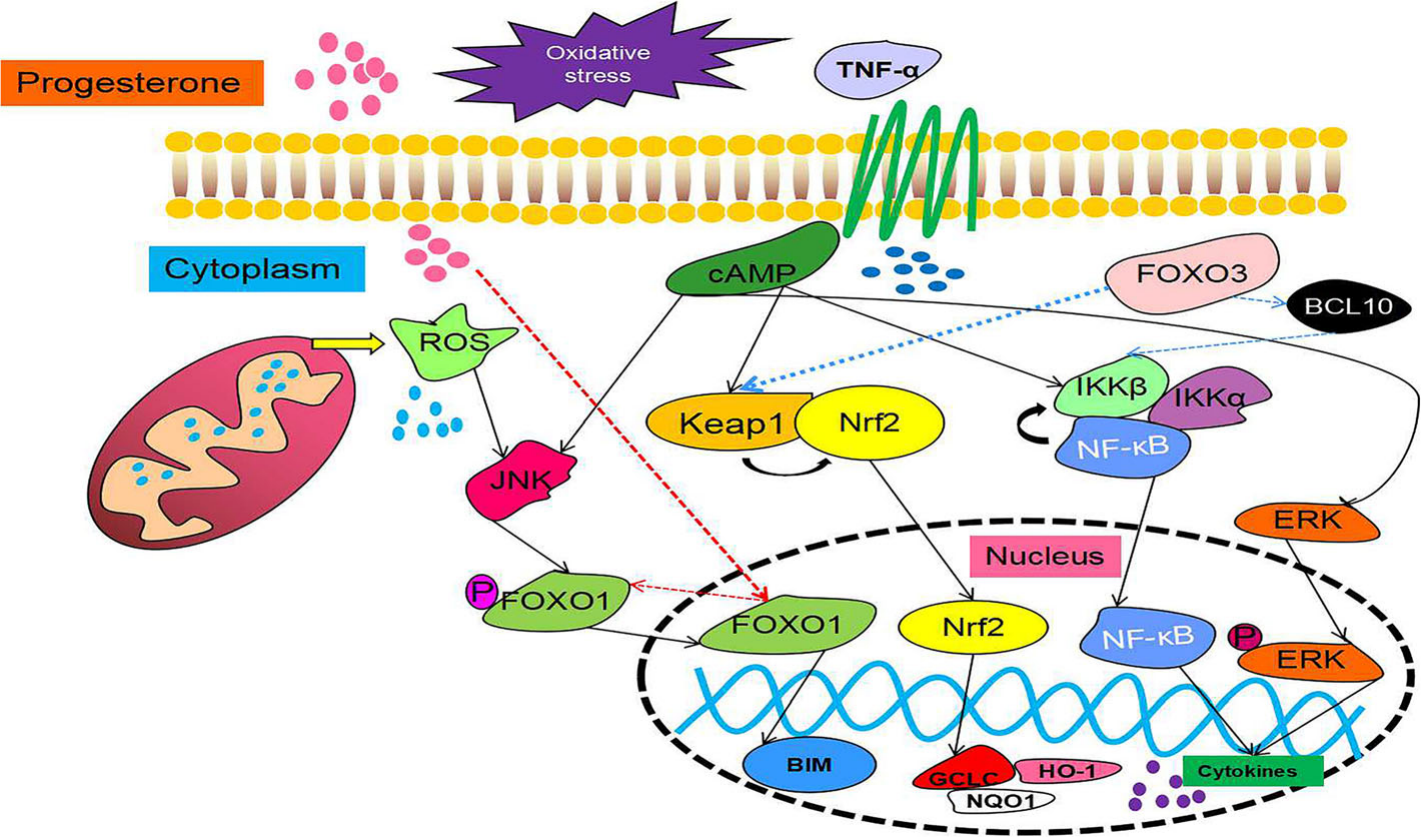
The signaling pathway of OS and pregnancy (a brief view). When the body, especially the maternal body, suffers from an imbalance between oxidation and antioxidant levels during pregnancy, in addition to changes in TNF-α, changes in progesterone cannot be ignored. First, TNF-α activates a series of signaling pathways in cells through cAMP, such as stimulation of the Keap1-Nrf2 signaling pathway, NF-κB signaling pathway, MAPK signaling pathway, etc., then promoting an increase in cytokines and changes in antioxidant-related genes. However, FOXO3 is involved in these signaling pathways. When FOXO3 is increased, it promotes the binding of Keap1-Nrf2, lowering the level of antioxidants and promoting the release of NF-κB by IKKβ by stimulating BCL10, thereby promoting the increase in cytokines and apoptosis. Finally, the mechanism underlying the changes in the FOXO family under the combined effects of both reproductive and oxidative stress remains unclear. It can only be demonstrated that JNK undergoes dephosphorylation of FOXO1 under the action of cAMP and ROS when oxidative stress occurs to induce it to enter the nucleus and promote apoptosis. When progesterone is reduced, nuclear translocation occurs in FOXO1, and it is phosphorylated

Further, FOXO1 is indispensable for the induction of the most highly responsive decidual marker genes, including WNT4, prolactin (PRL) and insulin-like growth factor-binding protein 1 (IGFBP1) [116]. It has been found that FOXO1 activates apoptotic pathways in decidual stromal cells. The pro-apoptotic Bcl-2 homology 3 domain-only protein BIM is a major intermediate in this pathway [117]. It was validated that BIM is a FOXO1 target gene and is induced under the stimulation of cAMP. Both cAMP and progestin promote increases in FOXO1, but BIM is only increased and cell death occurs when progestin disappears [120]. Additionally, targeted phosphorylation of cytoplasmic FOXO factors by JNK promotes nuclear import and increases cellular protection against OS via the transcriptional activation of MnSOD and CAT [118]. Thus, FOXO1 has emerged as a major regulator of progesterone-dependent differentiation of human endometrium and subsequent process (Fig. 7) [119]. Thoughtfully, FOXO1 is markedly induced upon decidualization both in vivo and in vitro, whereas FOXO3 expression is suppressed [120]. At any rate, FOXO1 plays a unique role either in reproduction or in OS (Table 2).

The extracellular environment activates three pathways, with ERK predominantly activated by inflammation and growth factors, while JNK and p38 MAPK are predominantly activated by stress and inflammation [121]. Additionally, studies by Lee et al. showed that ROS generated by dysfunctional electron transport in mitochondria activate the inflammatory ASK1-P-p38 MAPK pathway [122]. The recent literature has identified the physiologic aging of fetal tissues as a potential mechanistic feature of normal parturition. This process is affected by telomere-dependent and p38 MAPK-induced senescence activation (Fig. 7). Pregnancy-associated risk factors can cause pathologic activation of this pathway, causing OS-induced p38 MAPK activation and leading to senescence and premature aging of fetal tissues [123, 124].

It has been reported that the activation of ERK was increased in endometriotic tissue, suggesting that ERK might play a role in endometriosis pathogenesis, and phosphorylated ERK is increased in primary eutopic epithelial cells [125, 126]**.** Prolonged phosphorylation of ERK in endometrial stromal cells occurs in women with endometriosis, compared with women without endometriosis [127]. Therefore, the endometriotic microenvironment could induce increased ERK activity in ectopic cells. Although only IL-1β-induced cyclo-oxygenase 2 (COX2) production and IL-8 secretion could be attenuated by the ERK1/2-specific inhibitor PD98059, both TNF-α and IL-1β activate ERK and induce the expression of IL-8 and IL-6 [128]. However, another study found that the IL-1β-mediated COX2 expression was not affected when ERK inhibition occurs in endometriotic stromal cells, but it occurred rather through p38 MAPK activation [129]. OS may also contribute to ERK activation. H_2_O_2_ induces ERK phosphorylation in endometriotic stromal cells with a more serious induction compared with stromal cells from women who do not have endometriosis [130]. Today, although, no direct relationship between phosphorylated ERK (p-ERK) activation and OS is confirmed, an increase in OS markers is discovered in epithelial and stromal cells derived from women with endometriosis in a similar pattern to p-ERK level (Table 2).

## Conclusion

Based on the above, OS influences the entire reproductive process of woman. The production of excessive ROS leads to OS events. ROS, including superoxide (O_2_^−^•), hydrogen peroxide (H_2_O_2_) and hydroxyl (•OH), cause DNA damage, lipid per-oxidation and protein damage. The antioxidative system is activated when slight OS occurred, such as SOD and GPx. In addition, when ROS levels exceed the scavenging capacity of the system, the redox system can repair oxidized and damage molecules using NADPH as an original electron source in such situations. Thus, the maintenance of high redox potential is a prerequisite for maintaining the reproductive systems in a healthy state [15].

In this review, we mainly introduced the relative reproductive diseases caused by OS and a series of signaling pathways, including in PCOS, endometriosis, preeclampsia and so on. They switch on a variety of molecules, including NF-κB, MAPK, FOXO and Keap1-Nrf2. In the above descriptions, we found that the role of each molecule is not independent and that they will form networks and interactions between them, leading to the complexity of signal molecule research. The OS that occurs during reproduction activates many molecules, but the interaction among them is not very clear, requiring us to determine the signaling cues in other organs or other diseases.

It is speculated that we know that the FOXO protein family is involved in the signaling pathways of Keap1-Nrf2 and NF-κB. There are also subtle relationships between the various subtypes of the MAPK family. Therefore, regarding OS in the reproductive process, we can verify these viewpoints to better address the relationship between OS and reproductive harm. A large part of reproductive disease is caused by inflammation in the reproductive organs, leading to changes in the inflammatory factors that promote the body’s protection or, in severe cases, promote cell death.

Compared to other diseases, disease research in the reproductive system is complicated, especially in humans or females during pregnancy. This complexity requires us to concretize the experimental period and break it down. In this discussion, we summarize the following. First, OS is involved in the development of diseases of the reproductive system, and it plays the role of a double-edged sword. Second, to a large extent, OS impairs the reproductive organs, including the placenta. Third, the inflammatory environment caused by OS causes a series of signal activations in the uterus. Fourth, the connection between OS and progesterone causes the reproductive process to become obstructed. Finally, we can re-examine the future development trends in reproductive system diseases by speculating on the relationship between these signaling molecules.

Female animals also undergo complex reproductive changes during the course of their illnesses and their deaths. In this review, the follicular development of animals, the development of fertilized eggs, and the processes of hormone changes are demonstrated. OS-related reproductive diseases have also been elucidated. By speculating on the changes in other diseases and on the factors related to reproductive diseases in cells in the face of reproductive system diseases, this article provides some institutional recommendations. This common point of these signals is that they are activated during inflammatory processes induced by OS. Under the influence of TNF-α, cAMP messengers are activated, causing massive release of ROS in the mitochondria and deposition in the cytoplasm. Further, NF-κB, FOXO, and the MAPK family are induced. IKKβ releases NF-κB into the nucleus, resulting in a large number of cytokines increasing and promoting apoptosis. Further, FOXO3 directs the activation of BCL10, thus controlling NF-κB activity, but it is also activated through AKT pathway. ROS activate JNK to target phosphorylated FOXO1 in the cytoplasm to promote transcription into the nucleus. Progesterone’s antiproliferative effect on uterine epithelial cells is affected by FOXO1, and an increase in progesterone activates FOXO1 to release into the nucleus. Under the influence of TNF-α and IL-1β, the MAPK family is activated. When ERK is inhibited, p38 MAPK joins the battle, playing the same role as ERK.

In addition, Nrf2 plays an important role in mitigating OS-induced cellular dysfunction and developmental defects. Continued exposure to OS in postnatal and later life periods can further exacerbate the loss of Nrf2-regulated antioxidant defenses established in utero and thereby enhance susceptibility to disease in offspring. The Nrf2 antioxidant defense pathway might therefore provide a therapeutic target for ameliorating OS associated with adverse pregnancies and could provide an opportunity to modulate developmental priming via OS.

Although Nrf2 is a master regulator of cellular redox homeostasis following stress, Nrf2 activity in vascular and other cell types is known to decline [131] and could play an important role in age-related cellular dysfunction and disease onset. As highlighted by Zhang et al. in a special issue [132], the molecular mechanisms underlying the loss of response to OS by the Keap1-Nrf2 defense pathway in aging remain to be elucidated. Keap1-Nrf2 is the most classical pathway in OS, and it has also been shown in recent studies to play an important role in fetal development.

In conclusion, FOXO, as a key node of the signaling pathway, plays an important role in the signaling network and is a factor worth studying.

### Future directions

In the future, a strategy to reinforce the antioxidant defense system and target the mitochondria will be a huge step. To increase the antioxidant capacity of the body, we must decrease the production of ROS from the mitochondrial electron transport chain that occurs in response to high glucose and fatty acid levels and decrease ROS production without significantly affecting ATP production. At the same time, we should increase the degradation of intracellular ROS and increase the bioavailability of antioxidants, and their passage through the barriers must be considered. Targeting the mitochondria and increase its overall antioxidant defense system will be a challenge. It is now considered certain that the pharmacological effects of antioxidants depend on their targeting. The delivery of antioxidants to mitochondria is a field of active research [133].

In addition, if these signaling molecules were studied completely, we would develop many blocking agents to prevent the occurrence of damage. ROS-activated JNK molecules and their downstream FOXO transcription factors (also involved in reproductive events) are worth exploring. In addition, OS-induced NF-κB signaling molecules should be linked to the molecules of the reproductive process, and for the future better study of reproductive diseases, drugs have very important research value. As we all know, an increasing number of social diseases attack our bodies, and some aging questions also perplex us. The mechanisms described in this review have important implications for these diseases, and the silence of certain factors in the FOXO family can cause activation of the antioxidant mechanism, the role of which in tumor diseases cannot be underestimated, so the study of OS in the study of cell protection mechanisms is unique and critical for the mechanism of disease.

## Abbreviations

8-OHDG: 8-hydroxy-deoxyguanosine
BCL10: B-cell lymphoma/leukemia 10
CAT: Catalase
CL: Corpus luteum
CORT: Corticosterone
COX2: Cyclo-oxygenase 2
Cu-Zn SOD: Copper-zinc SOD
ERK1/2: Extracellular regulated kinase 1/2
ETC: Electron transport chain
FSH: Follicle-stimulating hormone
GCLC: Glutamate-cysteine ligase subunit catalysis
GPx: Glutathione peroxidase
GSR: Glutathione reductase
HCG: Human chorionic gonadotropin
HO-1: Heme oxygenase 1
IGFBP1: Insulin-like growth factor-binding protein 1
IKK: Inhibitor of nuclear factor kappa-B kinase
IUGR: Intrauterine growth restriction
JNK: Jun N-terminal kinase
Keap1: Kelch-like ECH-associated protein 1
MAPK: Mitogen-activated protein kinase
MCP-1: Monocyte chemoattractant protein-1
MDA: Malondialdehyde
MIF: Migration inhibitory factor
MnSoD: Manganese superoxide dismutase
NAC: N-acetylcysteine
NADP(H): Nicotinamide adenine dinucleotide phosphate
NF-κB: Nuclear factor-kappa B
NQO1: NAD(P)H dehydrogenase (quinone 1)
OS: Oxidative stress
ox-LDL: Oxidized LDL
PCOS: Polycystic ovary syndrome
PGF_2*α*_: Prostaglandin-F-2*α*
PRL: Prolactin
SO: Superoxide
SOD: Superoxide dismutase
TAS: Total antioxidant status
TNF-α: Tumor necrosis factor-α
uNK: Uterine natural killer
VEGF: Vascular endothelial growth factor
